# Intravitreal brimonidine inhibits form-deprivation myopia in guinea pigs

**DOI:** 10.1186/s40662-021-00248-0

**Published:** 2021-07-14

**Authors:** Yifang Yang, Junshu Wu, Defu Wu, Qi Wei, Tan Zhong, Jun Yang, Xiaowei Yang, Meizhen Zeng, Xingwu Zhong

**Affiliations:** 1 State Key Laboratory of Ophthalmology, Zhongshan Ophthalmic Center, Sun Yat-sen University, Guangzhou, 510060 China; 2 Hainan Eye Hospital and Key Laboratory of Ophthalmology, Zhongshan Ophthalmic Center, Sun Yat-sen University, Haikou, 570311 Hainan Province China

**Keywords:** Brimonidine, Myopia, Form deprivation, Retina

## Abstract

**Background:**

The use of ocular hypotensive drugs has been reported to attenuate myopia progression. This study explores whether brimonidine can slow myopia progression in the guinea pig form-deprivation (FD) model.

**Methods:**

Three-week-old pigmented male guinea pigs (*Cavia porcellus*) underwent monocular FD and were treated with 3 different methods of brimonidine administration (eye drops, subconjunctival or intravitreal injections). Four different concentrations of brimonidine were tested for intravitreal injection (2 μg/μL, 4 μg/μL, 20 μg/μL, 40 μg/μL). All treatments continued for a period of 21 days. Tonometry, retinoscopy, and A-scan ultrasonography were used to monitor intraocular pressure (IOP), refractive error and axial length (AL), respectively. On day 21, guinea pigs were sacrificed for RNA sequencing (RNA-seq) to screen for associated transcriptomic changes.

**Results:**

The myopia model was successfully established in FD animals (control eye vs. FD eye, respectively: refraction at day 20, 0.97 ± 0.18 D vs. − 0.13 ± 0.38 D, F = 6.921, *P* = 0.02; AL difference between day 0 and day 21, 0.29 ± 0.04 mm vs. 0.45 ± 0.03 mm, F = 11.655, *P* = 0.004). Among the 3 different brimonidine administration methods, intravitreal injection was the most effective in slowing myopia progression, and 4 μg/μL was the most effective among the four different concentrations of brimonidine intravitreal injection tested. The AL and the refraction of the brimonidine intravitreal injection group was significantly shorter or more hyperopic than those of other 2 groups. Four μg/μL produced the smallest difference in AL and spherical equivalent difference values. FD treatment significantly increased the IOP. IOP was significantly lower at 1 day after intravitreal injections which was the lowest in FD eye of intravitreal injection of brimonidine. At day 21, gene expression analyses using RNA-seq showed upregulation of *Col1a1* and *Mmp2* expression levels by intravitreal brimonidine.

**Conclusions:**

Among the 3 different administration methods, intravitreal injection of brimonidine was the most effective in slowing myopia progression in the FD guinea pig model. Intravitreal brimonidine at 4 μg/μL significantly reduced the development of FD myopia in guinea pigs. Expression levels of the *Col1a1* and *Mmp2* genes were significantly increased in the retinal tissues of the FD-Inj-Br group.

**Supplementary Information:**

The online version contains supplementary material available at 10.1186/s40662-021-00248-0.

## Synopsis

Among the 3 different administration methods (eye drops, subconjunctival or intravitreal injections), intravitreal injection of brimonidine was the most effective in slowing myopia progression in the form-deprivation guinea pig model. Intravitreal brimonidine at 4 μg/μL significantly reduced the development of form-deprivation myopia in guinea pigs. Expression levels of the *Col1a1* and *Mmp2* genes were significantly increased in the retinal tissues of the brimonidine intravitreal injection group.

## Highlights

We evaluated the effects of 3 different forms of brimonidine administration and 4 different concentrations of brimonidine delivered via intravitreal injection in attenuating form-deprivation myopia in guinea pigs and found that axial growth and refraction difference value were significantly reduced in eyes receiving brimonidine intravitreal injection. We also investigated the molecular mechanisms underlying the attenuation of myopia development using RNA-seq and found that *Col1a1* and *Mmp2* expression levels were upregulated by brimonidine treatment.

## Background

Myopia is a common public health problem that has far-reaching consequences and enormous potential economic impact [1, 2]. Anatomically, myopia is characterized by the excessive axial elongation of the eye [3]. Myopia that begins during middle childhood age is commonly known as school myopia [3]. As school myopia progresses to > − 6.0 diopters (D) or an ocular axial length (AL) ≥ 26.0 or 26.5 mm, high myopia occurs. The presence of posterior staphyloma, which is the most common finding in patients with pathologic myopia, is the key differentiating factor between high and pathologic myopia [4]. The occurrence of staphyloma will, in most cases, eventually lead to other conditions, such as atrophic, traction or neovascular maculopathy [4], while high myopia itself can cause retinal detachment, cataract and increased risk of glaucoma [5].

Clinical studies have shown that low-concentration 0.01% atropine eye drops can effectively slow the progression of myopia [6, 7]. Atropine is generally believed to block the development of myopia by interacting with muscarinic acetylcholine receptors [8]. However, recent studies have shown that at the concentrations used to inhibit myopia in chicks and tree shrews, either the α_1A_-, α_1D_-, or α_2A_-adrenergic receptors might be the “true” intermediate receptors involved in atropine inhibition of myopia. This idea is based on a few different pieces of evidence. First, in Luft’s study, 18 different muscarinic antagonists were injected into goggled chick eyes to test whether they were effective at inhibiting myopia. Although Luft’s group identified another muscarinic antagonist, oxyphenonium, that prevents form-deprivation myopia (FDM), it was surprising that so many of the other antagonists were rather ineffective; 15 out of 18 tested had either a partial effect, were ineffective or toxic [9]. McBrien’s study demonstrated that the highly selective allosteric M_4_ muscarinic receptor antagonist MT-3 is effective at significantly inhibiting FDM in chicks and attributed this effect to mAChR M4 [8]. However, it has been reported by Näreoja that MT-3 also has modest to high-affinity for α_1A_-, α_1D_- or α_2A_-adrenoceptors [10]. Recently, Carr et al. demonstrated that MT-3, and other effective myopia-inhibiting mAChR antagonists atropine, pirenzepine and oxyphenonium, bound to the ADRA2A receptor with affinities that were sometimes lower than those for the chick mAChR M_4_ receptor, and these affinities matched the concentrations of drug required to inhibit myopia in chicks as reported by Luft et al. [11]. Carr’s group also tested the hypothesis that α-adrenoceptors might be involved in the regulation of eye growth in chicks by intravitreal injection of various ADRA2A agonists (clonidine, guanfacine and brimonidine) and an antagonist (yohimbine). They found that high concentrations of α_2_-adrenoceptor agonists, like those required by atropine, inhibited FDM in chicks [12]. Considering these, a strong possibility is that cholinergic receptors in the retina are not the main targets through which atropine and other muscarinic antagonists may act to prevent FDM.

Brimonidine, an α_2_-adrenergic agonist, has been reported in preliminary studies to inhibit the development of lens-induced myopia in mammal guinea pigs and FDM in non-mammal chicks [12, 13]. However, the mechanism of action of drugs varies among different species, and the mechanisms of lens-induced and FDM might be sufficiently different for them to respond differently to this agonist in the same species [14]. Therefore, we chose to investigate the action of brimonidine and associated transcriptomic changes in guinea pig eyes with FDM myopia. Further exploring the effectiveness of brimonidine as a treatment for myopia and of different routes of administration in a mammalian animal model could lead to the discovery of better pharmacotherapeutic targets for the prevention and treatment of human myopia.

## Materials and methods

### Animal housing, ethics statement and experimental procedure

Three-week-old male pigmented guinea pigs (Changsha Tianqin Biotechnology Corporation, Changsha, China) were housed at the Sun Yat-sen University Laboratory Animal Centre at 24 °C, on a 12:12 light-dark cycle with food, water and additional fresh vegetables provided ad-libitum. Animal use protocols were approved by the Sun Yat-sen University Institutional Animal Care and Use Committee and followed the ARVO statement for the use of animals in ophthalmic and vision research.

A total of 130 animals were used in this study. All but 8 control animals were treated to induce FDM development in the right eye, and the same eye also underwent one of 10 treatments: form-deprivation (FD) only, eye drops (one drop, twice a day) of 4 μg/μL brimonidine or sterile water, subconjunctival injection of 4 μg/μL brimonidine or sterile water, or intravitreal injection of 2 μg/μL, 4 μg/μL, 20 μg/μL or 40 μg/μL brimonidine or sterile water. The animals received the treatments and FD induction simultaneously. Brimonidine tartrate (UK 14,304 tartrate; Sigma-Aldrich, St. Louis, Missouri, USA) was dissolved in sterile water at room temperature. Solutions were freshly prepared on the day of injection. A detailed flow chart can be found in Supplementary Fig. 2. First, we evaluated 16 animals in total from the two groups (FD group and control group, *n* = 8 each group) to test whether the FD model was successfully established. The FD group underwent FD in the right eye, while the control group did not. AL measurements were taken every 7 days and spherical equivalent (SE) measurements were taken every 10 days for 21 days, while intraocular pressure (IOP) was tested every two days for 21 days. Then, compared with the above FD group, we divided 48 animals into six treatment groups (eye drops of 4 μg/μL brimonidine or sterile water, subconjunctival injection of 4 μg/μL brimonidine or sterile water, intravitreal injection of 4 μg/μL brimonidine or sterile water, *n* = 8 each group) to examine the most effective brimonidine administration method. In this experiment, 48 animals underwent FD in the right eye. Eye drops were administered twice a day, one drop each time, while injections were administered every 4 days with 5 μL solution during the treatment period of 21 days, starting on day 0. AL was tested every 7 days for 21 days, while SE was tested every 10 days for 21 days. After that, we divided 30 animals into 5 groups (FD only, 2 μg/μL, 4 μg/μL, 20 μg/μL, 40 μg/μL, *n* = 6 each group) to evaluate the most effective concentration of intravitreal brimonidine. All 30 animals underwent FD in the right eye. Injections were administered every 4 days with 5 μL solution. AL and SE were tested every 10 days for 21 days. Finally, we divided 36 animals into 3 groups: FD, 4 μg/μL intravitreal injection of brimonidine and intravitreal injection of water (FD-No-Inj, FD-Inj-Br and FD-Inj-Wa, *n* = 12 per group). Injections were administered every 4 days with 5 μL solution. AL and SE were examined every week for 21 days, while IOP was tested every 2 days for 21 days. We compared only the right eyes of each group to avoid the potential influence of injections on the uninjected eyes.

### Induction of form-deprivation myopia and injections

Circular Velcro rings covered with a diffuser were attached to the periocular fur of the right eyes of animals under anesthesia by intraperitoneal injection of 2% pentobarbital sodium (30 mg/kg). Animals were monitored hourly during the 12-h-light period to ensure that the diffusers remained in place. The left eyes were left without diffusers. For injections, 5 μL of solution was injected into the right vitreous chamber or subconjunctival area using a microsyringe (Hamilton 700, 25 μL, Reno, USA) and bevelled needle (33 G) under the same anesthesia procedure. Injections were administered every 4 days from day 0; contralateral eyes were not injected.

### Optical and biometric measurements

IOP, SE, and optical AL were measured in both eyes of restrained animals without anesthesia before the initiation of FD treatment (baseline), with follow-up measurements at two-day (IOP) or approximately weekly (SE and AL) intervals over 21 days. Only data from right eyes are reported here. IOP and AL were measured after topical anesthesia with 4% oxybuprocaine hydrochloride (Santen Pharmaceuticals, Osaka, Japan).

All IOP measurements were conducted prior to other procedures with rebound tonometry (iCare; Tonolab, Helsinki, Finland) around the same time of the day, early in the morning, after lights-on. Five measurements were taken for each eye, and the average was used for data analysis.

Refractive errors were measured using streak retinoscopy on awake animals in a dark room 30 min after instillation of 3 drops of 1% tropicamide phenylephrine ophthalmic solution (Saten, Osaka, Japan) at 5-min intervals to induce cycloplegia. SE values were calculated as the averages of results for the two principal meridians for use in data analyses.

Ocular axial dimensions were measured by A-scan ultrasonography with a 25-MHz probe (AXIS-II; Quantel Medical Inc., Clermont-Ferrand, France) assuming the velocity of sound in the anterior chamber was 1557.5 m/s, 1723.3 m/s in the lens, and 1540 m/s in the vitreous chamber [15]. For each measurement, at least five traces were captured per eye and analyzed offline. Only optical ALs are reported here, derived as the sum of anterior chamber depth, axial lens thickness and vitreous chamber depth.

### RNA-seq and bioinformatics analysis

At day 21 of treatment, the retinas of 2 guinea pigs from each FD-Inj-Br, FD-Inj-Wa and FD-No-Inj groups were randomly selected for RNA-seq. Total RNA was extracted, and Smart-Seq2 [16–18] was used to purify poly-A+ transcripts. High-throughput sequencing was performed using an Illumina HiSeqTM 2000 instrument (HiSeq Nova, Illumina, San Diego, California, USA). The RNA-seq reads were aligned to the guinea pig genome assembly (GCF_000151735.1_Cavpor3.0, https://www.ncbi.nlm.nih.gov/assembly/GCF_000151735.1) using HISAT2. StringTie was used to calculate read counts [19]. An average of 47 million mapped reads per library were generated. Genes and treatment-dependent differences in transcript amounts with *P* < 0.01 and log_2_(fold change) > 1 in the DEseq2 analysis of two RNA-seq biological replicates were taken to identify differentially expressed genes (DEGs). Ggplot2 was used to generate volcano plots, and pheatmap was used to generate heatmaps. We performed Gene Ontology (GO) analysis with ClusterProfiler input for upregulated DEGs that were more highly expressed in the FD-Inj-Br group than in the FD-Inj-Wa group (> 1-fold).

### Immunohistochemical analysis

The retinal tissue was placed in 4% PFA solution immediately after removing from the eyeball, then stored at 4 °C for 24 h. Then, the retinal tissue was embedded into paraffin sample blocks after dehydration, transparency, and paraffin immersion procedures. Slices with a thickness of 5 μm were obtained using a Leica RM2016 microtome. The slices were placed successively into xylene I for 15 min, Xylene II for 15 min, anhydrous ethanol I for 5 min, anhydrous ethanol II for 5 min, 85% alcohol for 5 min, 75% alcohol for 5 min and finally in distilled water. The tissue slices were placed in a repair box filled with EDTA antigenic repair buffer (pH 6.0) and then underwent antigen repair in a microwave oven; medium heat for 8 min was used until the solution came to a boil. Then, heating was stopped for 8 min and kept at medium-low heat for 7 min. This process was performed with caution, preventing excessive evaporation of the buffer that can dry the slices. After cooling at room temperature, the slices were placed in PBS (pH 7.4) and washed by shaking on a decolorization shaker 3 times for 5 min each. Slices were placed in 3% hydrogen peroxide solution (hydrogen peroxide: pure water, 1:9 ratio) and incubated at room temperature for 25 min in the dark. The slices were placed in PBS (pH 7.4) and washed by shaking on a decolorizing shaker 3 times for 5 min each. A circle was then drawn around the tissue with a hydrophobic marker. Then, the slices were blocked at room temperature for 30 min (10% normal rabbit serum was used as blocking buffer for primary antibody from goat host; 3% BSA for primary antibodies from other sources). The blocking buffer was removed gently thereafter. Primary antibody specific for *MMP2* (#11548283; Invitrogen; 1:200) or *Col1a1* (#1310–08; SouthernBiotech; 1:200) prepared in PBS at a certain concentration was placed on the section. The slices were placed flat in a dark box and incubated overnight at 4 °C. The next day, slices were placed in PBS (pH 7.4) and washed by shaking on a decolorization shaker 3 times for 5 min each. HRP-labeled secondary antibodies corresponding to the primary antibody were then added to cover the tissue in the ring, and the tissues were incubated at room temperature for 50 min. The slices were placed in PBS (pH 7.4) and washed by shaking on a decolorization shaker 3 times for 5 min each. After the sections were slightly dried, the newly prepared DAB solution was added into the ring, and the color developing time was controlled under the microscope. Color development (brown yellow) was terminated by flushing slices in tap water. The slices then were dyed with Harris Hematoxylin for approximately 3  min, washed with tap water, differentiated with 1% hydrochloric acid and alcohol for a few seconds, washed with tap water, returned to blue with ammonia, and washed again with running water. The slices were then successively placed into 75% alcohol for 6 min, 85% alcohol for 6 min, anhydrous ethanol I for 6 min, anhydrous ethanol II for 6 min, xylene I for 5 min and then dehydrated to transparency. The slices were removed from the xylene, left to dry slightly then sealed with neutral gum, and an IHC profiler was used to quantify the area of positive staining [20].

### Data analysis

SPSS 22 (IBM, NY, USA) software was used for data analysis. Data for treated and control eyes, as well as derived differences at different time points are reported as mean ± SEM. For multiple groups of data with repeated measurements, if the homogeneity test of variance was satisfied, two-way repeated measures ANOVA with a Bonferroni post hoc test was applied to longitudinal data. If the data did not meet the homogeneity test of variance, the Kruskal-Wallis test was used. *P* values are reported in Supplementary Tables 1, 2, 3, 4.

## Results

### Establishment and validation of the FDM guinea pig model

**Fig. 1:**
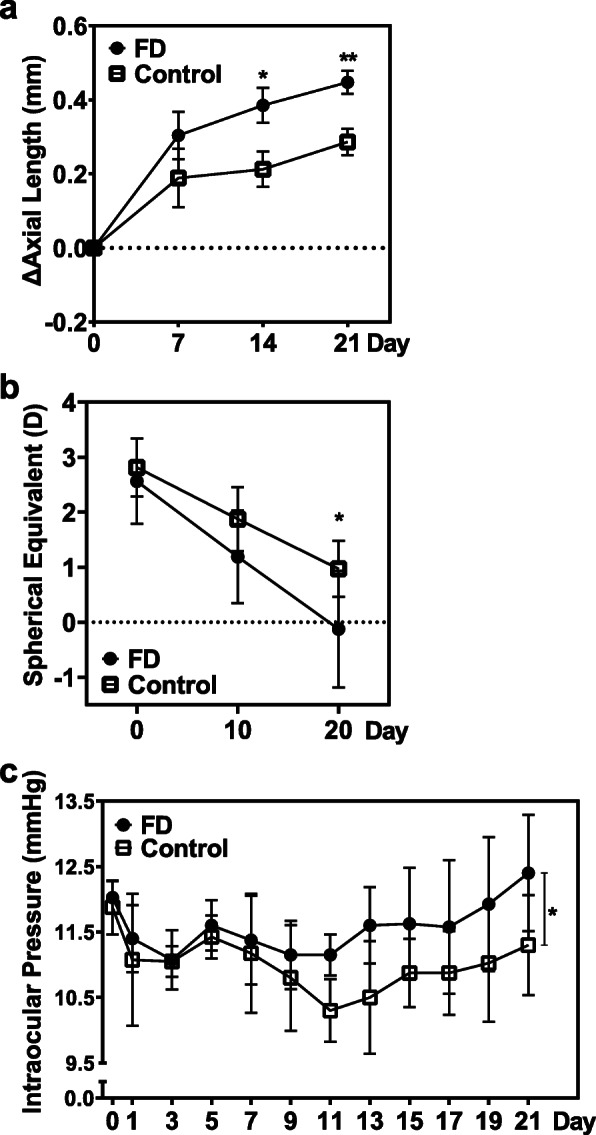
Alternation in axial length, refractive error and intraocular pressure in the form-deprivation (FD) group and control group. **a** Change in axial length (AL) difference value over the course of 3 weeks. **b** Change in spherical equivalent (SE) of refractive error difference over the course of 3 weeks. **c** Change in intraocular pressure (IOP) over the course of 3 weeks. *P < 0.05, **P < 0.005, (*n* = 8 each group). Only data from right eyes are reported here

The FD eyes showed significant ocular AL elongation and myopic shift in SE, as reflected in the progressive increase in each measurement and the difference values (i.e., the value of each measurement minus the value of the measurement on day 0) in FD eyes over the 21-day treatment period (Fig. 1a and b). As myopia progressed, the IOP in the FD myopic eyes gradually became higher than that in the control eyes (Fig. 1c). There were significant differences in IOP at time points before and after model establishment (F = 8.811, *P* = 0.01, Supplementary Table 1).

Statistically significant differences were found between the AL difference values at day 14 (control eye vs. FD eye, 0.21 ± 0.05 mm vs. 0.39 ± 0.05 mm, F = 6.521, *P* = 0.023) and day 21 (control eye vs. FD eye, 0.29 ± 0.04 mm vs. 0.45 ± 0.03 mm, F = 11.655, *P* = 0.004) and the SE value at day 20 (control eye vs. FD eye, 0.97 ± 0.18 D vs. − 0.13 ± 0.38 D, F = 6.291, *P* = 0.02). A statistical comparison between FD and control eyes is summarized in Supplementary Table 1. FD treatment significantly increased the IOP (control eye vs. FD eye, 11.02 ± 0.13 mmHg vs. 11.58 ± 0.13 mmHg, F = 5.456, *P* = 0.001).

### Intravitreal brimonidine effectively attenuated myopia

**Fig. 2:**
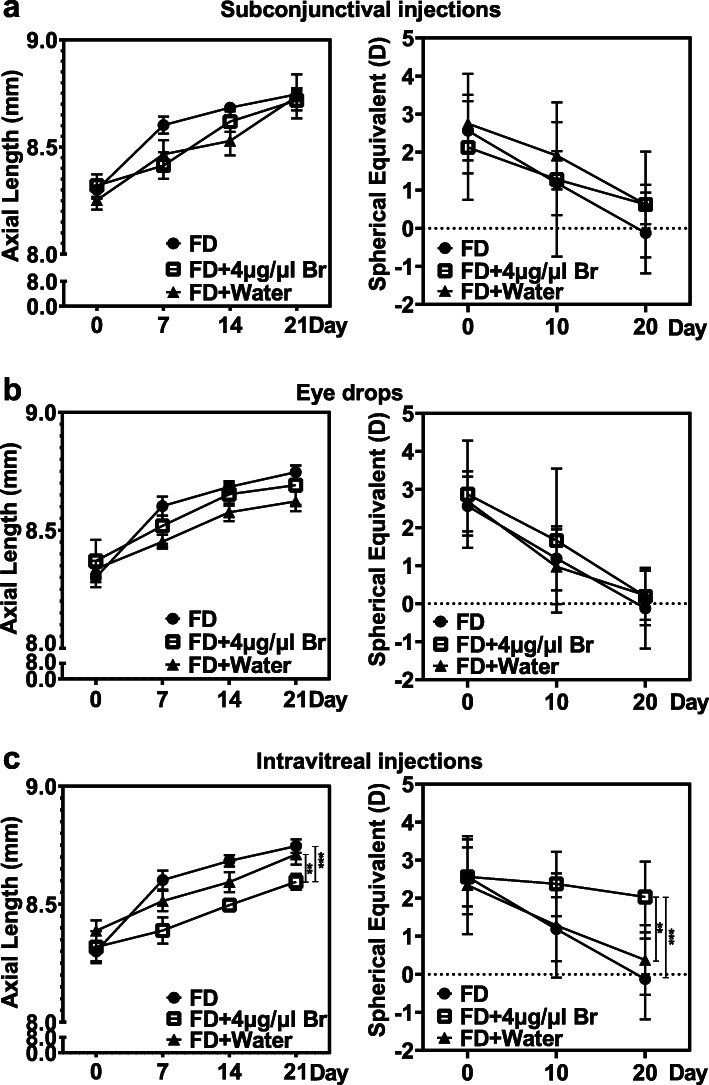
Efficacy of different brimonidine administration methods in slowing form-deprivation myopia. **a** Axial length (AL) and spherical equivalent (SE) of refractive error of the subconjunctival injection method. **b** AL and SE of the eye drop method. **c** AL and SE of the intravitreal injection method. **P < 0.005, ***P < 0.001 (*n* = 8 each group). Only data from right eyes are reported here

To evaluate the efficacy of 4 μg/μL brimonidine in attenuating progressing myopia, 3 administration methods, eye drops and subconjunctival or intravitreal injections, were applied to deliver brimonidine to the FD eyes of guinea pigs. Intravitreal injection of brimonidine was found to be more effective than intravitreal injection of water and no-treatment control, while eye drops and subconjunctival injection groups showed no significant effect (Fig. 2, Supplementary Table 2). The AL of the intravitreal injection of the brimonidine group was significantly shorter than that of the intravitreal injection water and no-treatment control groups (brimonidine intravitreal injection vs. water intravitreal injection vs. no-treatment, 8.45 ± 0.02 mm vs. 8.55 ± 0.02 mm vs. 8.58 ± 0.02 mm, F = 9.370, *P* = 0.001). The SE of the intravitreal injection of the brimonidine group was significantly hyperopic compared with that of the intravitreal injection water and no-treatment control groups (brimonidine intravitreal injection vs. water intravitreal injection vs. no-treatment, 2.32 ± 0.30 D vs. 1.33 ± 0.30 D vs. 1.21 ± 0.30 D, F = 4.148, *P* = 0.030).

**Fig. 3:**
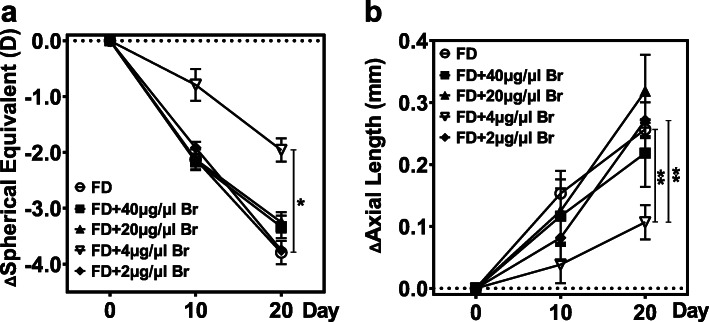
Efficacy of different concentrations of intravitreal brimonidine in slowing form-deprivation myopia. **a** Change in spherical equivalent (SE) of refractive error difference value over the course of 20 days. **b** Change in axial length (AL) difference over the course of 20 days. *P < 0.05, **P < 0.005, (*n* = 6 each group). Only data from right eyes are reported here

Among the 2 μg/μL, 4 μg/μL, 20 μg/μL and 40 μg/μL intravitreal brimonidine injections, 4 μg/μL was found to be the most effective in attenuating myopia progression in terms of AL elongation and SE myopic shift (Fig. 3, Supplementary Table 3). The 4 μg/μL group exhibited the shortest AL difference value at day 20 (2 μg/μL vs. 4 μg/μL vs. 20 μg/μL vs. 40 μg/μL, 0.27 ± 0.03 mm vs. 0.10 ± 0.02 mm vs. 0.32 ± 0.06 mm vs. 0.22 ± 0.05 mm, F = 4.238, *P* = 0.009) and the most hyperopic SE difference value at day 10 (2 μg/μL vs. 4 μg/μL vs. 20 μg/μL vs. 40 μg/μL, − 1.92 ± 0.11 D vs. − 0.79 ± 0.28 D vs. − 2.13 ± 0.19 D vs. − 2.17 ± 0.14 D, *P* = 0.005) and day 20 (2 μg/μL vs. 4 μg/μL vs. 20 μg/μL vs. 40 μg/μL, − 3.75 ± 0.06 D vs. − 1.96 ± 0.21 D vs. − 3.25 ± 0.18 D vs. − 3.33 ± 0.20 D, *P* = 0.001).

**Fig. 4:**
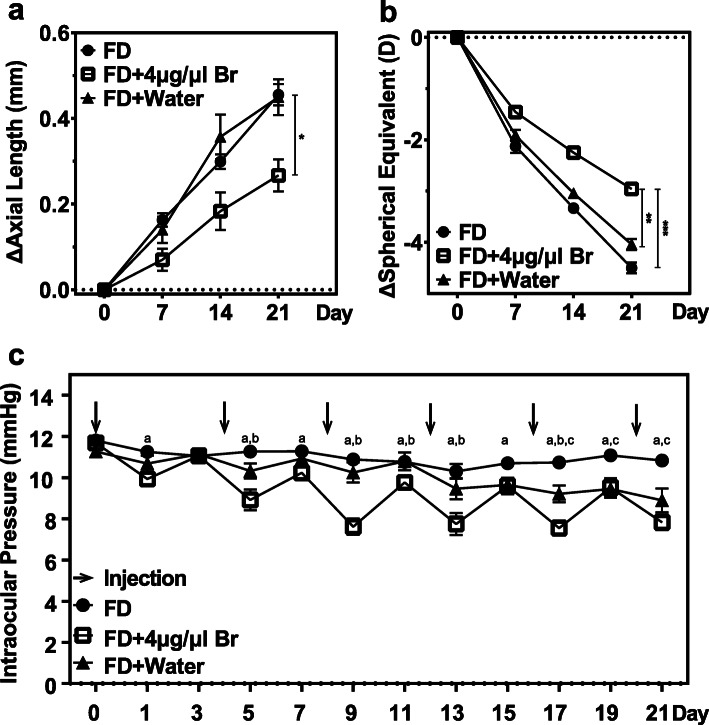
Efficacy of intravitreal injection of brimonidine in slowing form-deprivation myopia. **a** Change in axial length (AL) difference over the course of 3 weeks. **b** Change in spherical equivalent (SE) of refractive error difference over the course of 3 weeks. **c** Change in intraocular pressure (IOP) over the course of 3 weeks. *P < 0.05, **P < 0.005, ***P < 0.001 (*n* = 12 each group, ^*a*^ indicates a significant difference between FD + Br and FD at *P* < 0.05, ^*b*^ indicates a significant difference between FD + Br and FD + Water at *P* < 0.05, ^*c*^ indicates a significant difference between FD + Water and FD at *P* < 0.05). Only data from right eyes are reported here

Among the FD-Inj-Br, FD-Inj-Wa and FD-No-Inj groups, the FD-Inj-Br group showed much smaller changes in AL and SE difference values over the 21 days (AL difference values: FD-Inj-Br vs. FD-Inj-Wa vs. FD-No-Inj, 0.27 ± 0.04 mm vs. 0.45 ± 0.04 mm vs. 0.46 ± 0.04 mm, F = 9.149, *P* = 0.001; SE difference value: FD-Inj-Br vs. FD-Inj-Wa vs. FD-No-Inj, − 2.96 ± 0.32 D vs. − 4.04 ± 0.37 D vs. − 4.50 ± 0.37 D, *P* < 0.001, Fig. 4, Supplementary Table 4). IOP was variable but was significantly lower at 1 day after intravitreal injections, and lowest in the FD-Inj-Br group (FD-Inj-Br vs. FD-Inj-Wa vs. FD-No-Inj, 9.29 ± 0.20 mmHg vs. 10.17 ± 0.20 mmHg vs. 11.00 ± 0.20 mmHg, *P* < 0.001, Fig. 4c and Supplementary Table 4). Relevant mean baseline, AL and SE difference values and IOP data for the three groups are summarized in Supplementary Table 4.

**Fig. 5:**
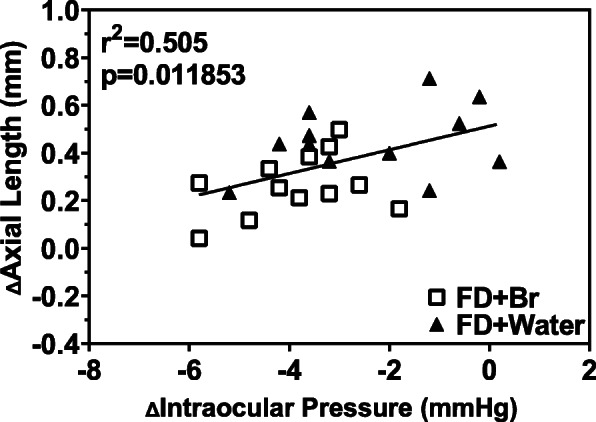
Regression analysis of axial length and IOP in the FD-Inj-Br and FD-Inj-Wa groups (r^2^ = 0.505, *P* < 0.05)

To explore the patterns of the changes in IOP in different treatment groups during FDM establishment, linear regression was performed on IOP data measured in FD eyes over 21 days. A regression analysis undertaken on FD-Inj-Br and FD-Inj-Wa data revealed a low-moderate linear correlation of AL with IOP (Fig. 5, r^2^ = 0.505, *P* < 0.05), providing indirect evidence for a role of IOP as an inflationary force in myopia development, which will be discussed further later.

### Differentially expressed genes (DEGs)

**Fig. 6:**
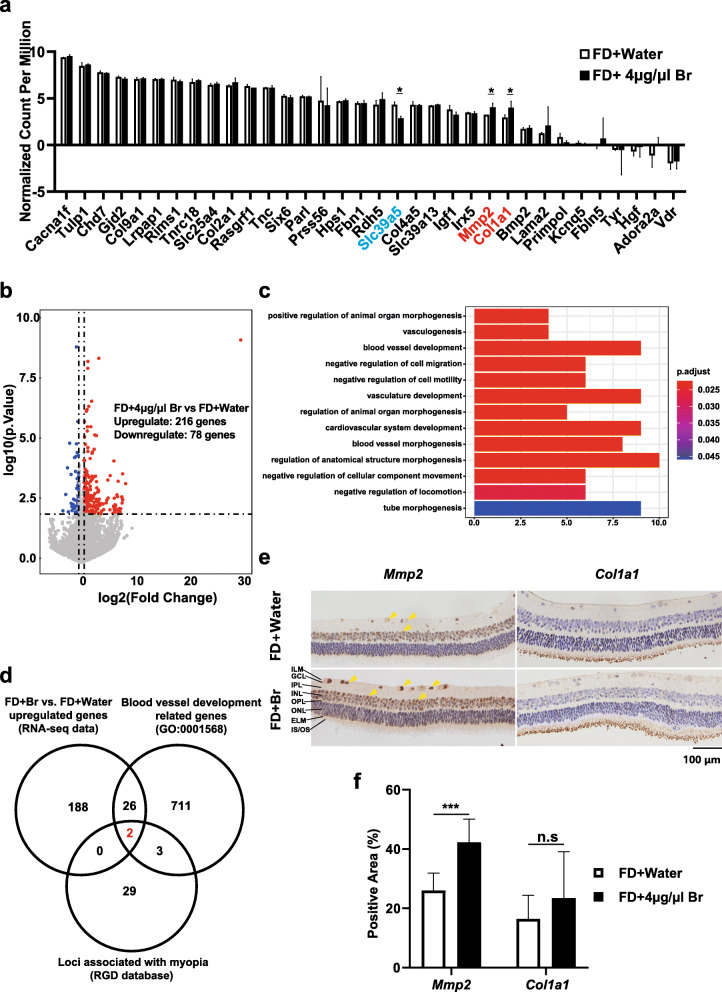
RNA-seq analysis of retina tissues from FD-Inj-Br and FD-Inj-Wa groups and IHC verification. **a** The expression of myopia-related genes in the FDM-Inj-Br group and FDM-Inj-Wa group. In the FDM-Inj-Br group, *Col1a1* and *Mmp2* expression levels were significantly increased, and *Slc39a5* expression level was significantly decreased (*P < 0.05). **b** Distribution of differentially expressed genes in the FDM-Inj-Br group and FDM-Inj-Wa group. Compared with the FDM-Inj-Wa group, 216 genes were upregulated in the FDM-Inj-Br group, and 78 genes were downregulated. **c** The functional enrichment of genes with increased expression after intravitreal injection of brimonidine was obtained through GO analysis. The results showed that among the top 10 most significant items, the items related to tissue regeneration were as follows: vasculogenesis, blood vessel development, and vasculature development. **d** Overlap with the myopia-related gene list. The genes significantly upregulated in FD-Inj-Br compared with FD-Inj-Wa and the gene sets involved in the biological process of tissue regeneration in the GSD and Mouse Genome Informatics (MGI) databases were compared by Venn diagram. **e** Immunohistochemical staining and analysis of retinal tissue in the FD-Inj-Br group and FD-Inj-Wa group. **f** The expression level of the Mmp2 protein in the retinal tissues of the brimonidine group was significantly increased compared with that in the water group (yellow triangle indicates Mmp2-positive signal), but there was no significant difference in the expression level of the Col1a1 protein

To explore gene expression in the retinal tissues of guinea pigs after intravitreal injection of brimonidine, transcriptome sequencing was performed on retinal tissues at day 21 of the experiment. We obtained retinal tissue with almost no choroid membrane (Supplementary Fig. 3 a). First, we explored the expression levels of genes annotated as related to myopia in the Rat Genome Database (RGD), database in retinal tissues of guinea pigs in the FD-Inj-Br and FD-Inj-Wa groups. The results showed that the expression levels of the *Col1a1* and *Mmp2* genes were significantly increased (*P* < 0.05) in the retinal tissues of the FD-Inj-Br group, and the expression level of *Slc39a5* was significantly decreased (*P* < 0.05), while there were no significant differences in the expression of other myopia-related genes between the two groups (Fig. 6a). Among the 25 adrenergic pathway-related genes, compared with the FD-Inj-Wa group, the *Pln* gene was significantly higher in the FD-Inj-Br group, the *Lmbrd2* gene was significantly lower in the FD-Inj-Br group, and the remaining 23 genes had no significant change. In the FD-Inj-Br group, compared with the FD-Inj-Wa group, 216 genes were upregulated, and 78 genes were downregulated (Fig. 6b, Supplementary Table 5). The functional enrichment of genes expressed at higher levels after intravitreal injection of brimonidine was obtained through GO analysis. The results showed that the upregulated genes were mainly enriched in terms related to angiogenesis, cell movement and migration and morphogenesis (Fig. 6c). In terms of overlap with the list of myopia-related genes, the genes upregulated in the FD-Inj-Br group showed significantly greater overlap than those upregulated in the FD-Inj-Wa group. Moreover, the upregulated genes were compared with the gene sets involved in the biological process of angiogenesis in the GSD and Mouse Genome Informatics (MGI) databases by Venn diagram (Fig. 6d); this analysis revealed two genes present in all three sets: *Col1a1* and *Mmp2* (Fig. 6d). Finally, we subjected the retinal tissue to immunohistochemical staining and analysis, and it was found that the expression levels of the *Mmp2* protein in the retinal tissue was significantly higher in the FD-Inj-Br group than in the FD-Inj-Wa group, while no difference in the expression of *Col1a1* was found between FD-Inj-Br and FD-Inj-Wa groups (Fig. 6e-f).

## Discussion

The main finding of this research is that intravitreal injection of brimonidine is effective in slowing the development of FDM in a mammalian model. In addition, brimonidine might affect the development of myopia by upregulating *Col1a1* and *Mmp2* gene expression in the retina.

To date, there have been 3 studies investigating the effects of intervention with adrenergic agonist drugs on myopia progression in animal models. However, the relationship between adrenergic receptors and myopia is still controversial. The three studies involved FD chickens, cell-receptor binding assays and lens-induced myopia in guinea pigs. The first two of the mentioned studies reported that muscarinic acetylcholine receptor (mAChR) antagonists block human α_2A_-adrenergic receptor (hADRA2A) signaling at concentrations comparable to those used to inhibit chick myopia *in vivo* and that high concentrations of α_2_-adrenoceptor agonists inhibited FDM in chicks [11, 12]. The latter of the three studies provided evidence that treatment with 0.1% brimonidine alone and 0.2% brimonidine alone, and not requiring the combination with pirenzepine, was effective in slowing the development of lens-induced myopia in guinea pigs, indicating that IOP elevation may be a promising mechanism for progressing myopia and suggesting potential treatment options [13].

The first question that this study sought to determine was whether brimonidine influences FD-induced myopia. The results of this study indicate that intravitreal injection of brimonidine can delay the progression of FDM in guinea pigs. One unanticipated finding was that not all modes of administration and not all concentrations of brimonidine are associated with delayed progression of myopia. Intravitreal injections of brimonidine were found to slow FDM progression, while eye drop instillation and subconjunctival injection did not. Moreover, a specific concentration of 4 μg/μL brimonidine was found to slow FDM progression, while 2 μg/μL, 20 μg/μL, and 40 μg/μL did not. The finding that only intravitreal injection can slow the progression of myopia is different from Liu’s published paper concerning lens-induced myopia (LIM) guinea pigs, in which eye drops were used [13]. In Liu’s study, 0.1% and 0.2% brimonidine were instilled into the guinea pig eye, one drop each time, twice a day, for 21 days, and they were effective at slowing the LIM in guinea pigs. One drop is approximately 20 μL; 0.1% concentration means 1 μL eye drop containing 1 μg brimonidine, while 0.2% means 1 μL eye drop containing 2 μg brimonidine. Thus, the 4 μg/μL concentration we used is equal to 0.1% brimonidine in Liu’s work which was effective at slowing the development of LIM in guinea pigs. However, the mechanisms of LIM and FDM might be sufficiently different to explain the difference in response. Similarly, it is also possible that the short duration of treatment in the animal model was insufficient and that significant differences might be observed for the other two methods of administration if longer treatment times were examined. In addition, this study suggested that there may be a “therapeutic window” of intravitreal brimonidine concentration. Since brimonidine was injected directly into the vitreous cavity, the drug was in direct contact with the retina. Here, we use the G protein switch-mediated negative feedback regulation model to explain the possible mechanism of brimonidine delaying the progression of FDM. In this model, we assume that there are three possible states when the cell experiences the direct or indirect action of brimonidine, namely, (i) full activation condition was not reached, (ii) activation was stronger than the negative feedback inhibition, or (iii) negative feedback inhibition was stronger than the activated state. When brimonidine does not reach a sufficient concentration to stimulate the cells, the downstream signaling pathways are not fully activated, so the lower concentration of brimonidine fails to delay the progression of myopia. When brimonidine reaches a certain therapeutic concentration, the cells on the retina are activated. Currently, although the mechanism of negative feedback inhibition also exists, it is not enough to exceed the activation effect, so the overall performance is a state where brimonidine plays a role in delaying the progression of myopia. However, if the dosage of brimonidine is further increased, the cells will receive a large amount of stimulation. At this time, the negative feedback inhibition regulation mechanism is activated, and the cells are in a state where the negative feedback inhibition is stronger than the activation, and thus a higher concentration of brimonidine does not delay the progression of myopia. We propose this hypothetical model based on the following three points: (i) We have observed that brimonidine only plays a role in delaying the progression of FDM at a certain concentration; (ii) Brimonidine is an α_2_-adrenergic receptor agonist, which is coupled to inhibitory Gi and Go proteins [21]; (iii) In G protein-coupled receptors, G protein conversion can mediate a negative feedback regulation mechanism [22]. In our model, when brimonidine is at a therapeutic concentration, G protein is activated to delay the progression of myopia. However, at higher concentrations, brimonidine may also mediate the activation of inhibitory G protein, thereby exerting inhibitory negative feedback regulation.

The common way to test whether a drug is associated with eye growth is by intravitreal injection, usually in extremely high concentrations, into the eyes of animal myopia models. The guinea pig is an ideal animal model for myopia research; its ocular structure is similar to that in humans, it has large pupils and reasonably large eyes, it is docile and cooperative, and its refraction and ocular biometrics are easily measured [23]. Therefore, our study aimed to further explore the hypothesis that α-adrenoceptors might be involved in the retinal regulation of eye growth in this powerful mammalian model.

Brimonidine, with effects on both aqueous inflow and uveoscleral outflow, is an ocular hypotensive drug. Previous studies on the correlation between IOP and myopia have produced inconsistent results. Some human studies have found no statistically significant difference between IOP in myopia and emmetropia [24], while others have found that IOP was significantly higher in severe myopes than in emmetropes and was correlated with the increase in AL [25]. Previous cross-sectional and prospective studies in children, addressing the question of whether IOP is related to myopia prevalence or progression have generally shown no such associations [24, 26, 27]. The SCORM (Singapore Cohort Study of the Risk Factors for Myopia) study found that IOP was not correlated with SE refraction and that there was no difference in pressure between refractive groups at baseline [24]. The Correction of Myopia Evaluation Trial (COMET) study reported that IOP was not associated with sex, baseline refractive error, baseline AL, myopia progression or change in AL, over the 5-year observation period [26]. Despite all these findings, it is well known that the elevated ocular pressure found in congenital glaucoma is associated with a higher rate of axial elongation in infant eyes [28]. It is still not known whether variations in the range of normal IOP could influence ocular growth and myopia development in school-age children. Furthermore, the results of a longitudinal experimental study of IOP in chicks under FDM or LIM were inconsistent [29].

The second aim of this study was to explore whether there is a correlation between IOP and myopia. In our study, we found a moderate positive relationship (r^2^ = 0.505, *P* < 0.05) between the change in IOP and AL, and FD eyes showed a tendency of elevated IOP, which is consistent with the possibility that structural changes in myopic (FD) eyes can lead to IOP elevation. The results of this study confirm that there is a positive correlation between changes in IOP and changes in the AL in the guinea pig FD model, which is consistent with the results of a previous study [30]. The FD treatment causes the retinal image to be blurred during the development of the eyeball. According to these data, we may infer that the image blurring may further cause an increase in IOP. To normalize the IOP, the developing eyeball may increase in volume to achieve a decrease in IOP, thereby causing the AL of the eyeball to become longer to relieve the increase in IOP.

Further studies of treated guinea pig retinas were performed by RNA-seq to identify candidates for key genes and pathways in myopia. The *Col1a1* and *Mmp2* genes have been reported to encode the extracellular matrix component collagen and are engaged in extracellular matrix remodeling in the sclera [31, 32]. In addition, the proteins encoded by *Col1a1* and *Mmp2* are involved in angiogenesis and the myopia pathway (among other activities) in mice and guinea pigs [33–36]. Here, we found that the expression levels of these two genes were upregulated in the retina after the intravitreal injection of brimonidine. Although the GO analysis results indicate that most of the upregulated genes are related to angiogenesis, the retina of guinea pig, as an avascular tissue, does not generate blood vessels. Therefore, it is speculated that the role of these angiogenesis-promoting genes in the retina may be to promote the regeneration of certain cells or tissues. There are reports suggesting that *Mmp2* is involved in retinal regeneration [37]. In addition, the genes up-regulated in the FD-Inj-Br group are enriched in the negative regulation of cell migration and morphogenesis-related terms, which suggests that the brimonidine-mediated delay in myopia progression may be associated with these biological functions. Further studies are needed to verify the mechanism by which brimonidine delays the progression of myopia. Furthermore, mutations in *SLc39a5* have been identified as being associated with pathogenic high myopia in a few studies [38–40]. *Slc39a5* encodes the solute carrier family 39 member 5, which is a key member of the ZIP transporters for metal ions, especially in mammalian zinc homeostasis [41]. The retina contains particularly high amounts of zinc, suggesting a pivotal role in the tissue [42]. Zn^2+^ dysregulation is a major factor limiting the survival and regenerative capacity of injured retinal ganglion cells (RGCs) [43]. In the FD-Inj-Br group, the expression of *Slc39a5* was significantly decreased, which was 1/78 of the genes that were significantly down-regulated. However, GO analysis of these genes showed that they were not enriched in the current guinea pig GO-related terms. Therefore, with the support of existing data, we did not conduct further verification or mining on *Slc39a5*. Among the 25 adrenergic pathway-related genes, compared with the FD-Inj-Wa group, the expression of two genes changed significantly in the FD-Inj-Br group. The *Pln* gene was significantly increased in the FD-Inj-Br group, and *Lmbrd2* gene was significantly reduced in the FD-Inj-Br group. Therefore, we speculate that brimonidine may not rely on the adrenergic pathway when delaying FDM.

The mechanism by which brimonidine delays FDM may act not only through IOP but also directly on the retina. Among the three modes of administration, intravitreal injection delivers the drug directly to the retina, and the effective concentration of the four concentrations is remarkably similar to that required to activate nerve receptors. Moreover, there are abundant retinal nerve receptors in the retina. Therefore, we hypothesize that brimonidine increases the expression of genes related to tissue regeneration in retinal tissues. These genes may improve the perception of blurred images in the retina in the FD model, thereby alleviating the occurrence of myopia. However, since the effect of image blurring cannot be eliminated, myopia cannot be suppressed, but only relieved.

With our research design, we are unable to strictly distinguish between actions on the adrenergic receptors and IOP. We found that intravitreal brimonidine could lower the IOP and slow myopia progression in FD eyes. RNA-sequencing data showed that at day 21, the expression of adrenergic signaling-related genes in the retina was not significantly different between the FDM-Inj-Br group and FDM-Inj-Wa group. Among the set of adrenergic signaling-related genes, 23 of 25 genes had no significant difference in expression between groups. This indicated that the effect of slowing myopia progression by brimonidine may not occur through an adrenergic signaling-related pathway. Moreover, we found no implications for the site of action of atropine.

It cannot be overlooked that as many as 20–25% of genes whose functions have not been determined might also play a role in myopia. Although the guinea pig is often used to study myopia, its genome has not been fully characterized, and further genetic studies are needed.

We must recognize that there are some shortcomings in our experiment. All the samples of guinea pig retinas were collected 21 days after the induction of FDM. Consequently, by design, our study focused on identifying the effects of FD rather than its causes, which might be detected after much shorter intervals after initiating FD. Therefore, further research should be conducted to explore early changes in gene expression during FD and to extend analyses to other tissues in the retina-to-sclera signal transmission chain. Combined with bioinformatics research, future studies can explore regulatory pathways or networks and locate key regulatory factors in myopia. In addition, we found that most of the guinea pigs lost their diffusers one to two times per week, although no longer than 1 h at a time, which may have had some unexpected influence on the results. Moreover, the intervals between the tested doses of brimonidine (2 μg/μL, 4 μg/μL, 20 μg/μL and 40 μg/μL) were quite broad and future studies may evaluate more doses than the present study.

## Conclusions

Among the three different administration methods (eye drops, subconjunctival injections, and intravitreal injections), brimonidine intravitreal injection was the most effective in slowing myopia progression in the FD guinea pig model. Intravitreal brimonidine (4 μg/μL) significantly reduced the development of FDM in guinea pigs. Expression levels of the *Col1a1* and *Mmp2* genes were significantly increased in the retinal tissues of the FD-Inj-Br group.

## Supplementary Information

**Additional file 1: Supplementary Figure 1.** Form-deprivation model of guinea pigs used in this study. Guinea pig wearing a diffuser on the right eye.

**Additional file 2: Supplementary Figure 2.** Flow chart of experimental design.

**Additional file 3: Supplementary Figure 3.** Labelled retinal layers (ILM, internal limiting membrane. NFL, nerve fiber layer. GCL, ganglion cells layer. IPL, inner plexiform layer. INL, inner nuclear layer. OPL, outer plexiform layer. ONL, outer nuclear layer. ELM, external limiting membrane. IS/OS, inner segment/outer segment). Adrenergic and cholinergic signaling-related gene set.

**Additional file 4: Supplementary Table 1.** Statistical analysis results of Fig. 1.

**Additional file 5: Supplementary Table 2.** Statistical analysis results of Fig. 2.

**Additional file 6: Supplementary Table 3.** Statistical analysis results of Fig. 3.

**Additional file 7: Supplementary Table 4.** Statistical analysis results of Fig. 4.

**Additional file 8: Supplementary Table 5.** RNA-seq analysis data.

## Data Availability

The datasets during and/or analyzed during the current study available from the corresponding author on reasonable request.
