# Supplementary figures and images for: Intravitreal brimonidine inhibits form-deprivation myopia in guinea pigs
Source: Eye Vis (Lond). 2021 Jul 14;8:27. doi: 10.1186/s40662-021-00248-0 (PMC8278638; doi:10.1186/s40662-021-00248-0)

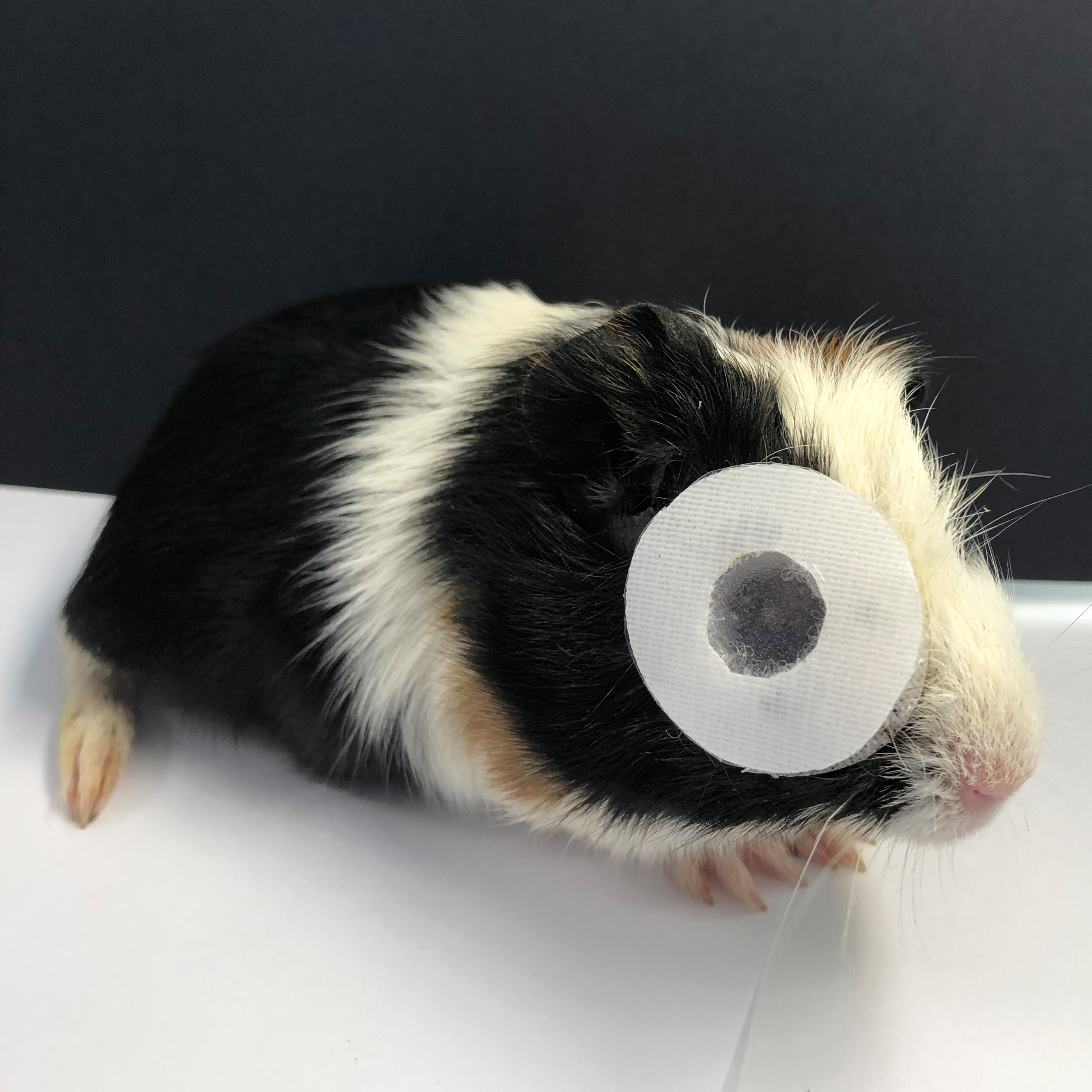

Supplement: Supplementary file 1 — Additional file 1: Supplementary Figure 1. Form-deprivation model of guinea pigs used in this study. Guinea pig wearing a diffuser on the right eye. [file 40662_2021_248_MOESM1_ESM.jpg]

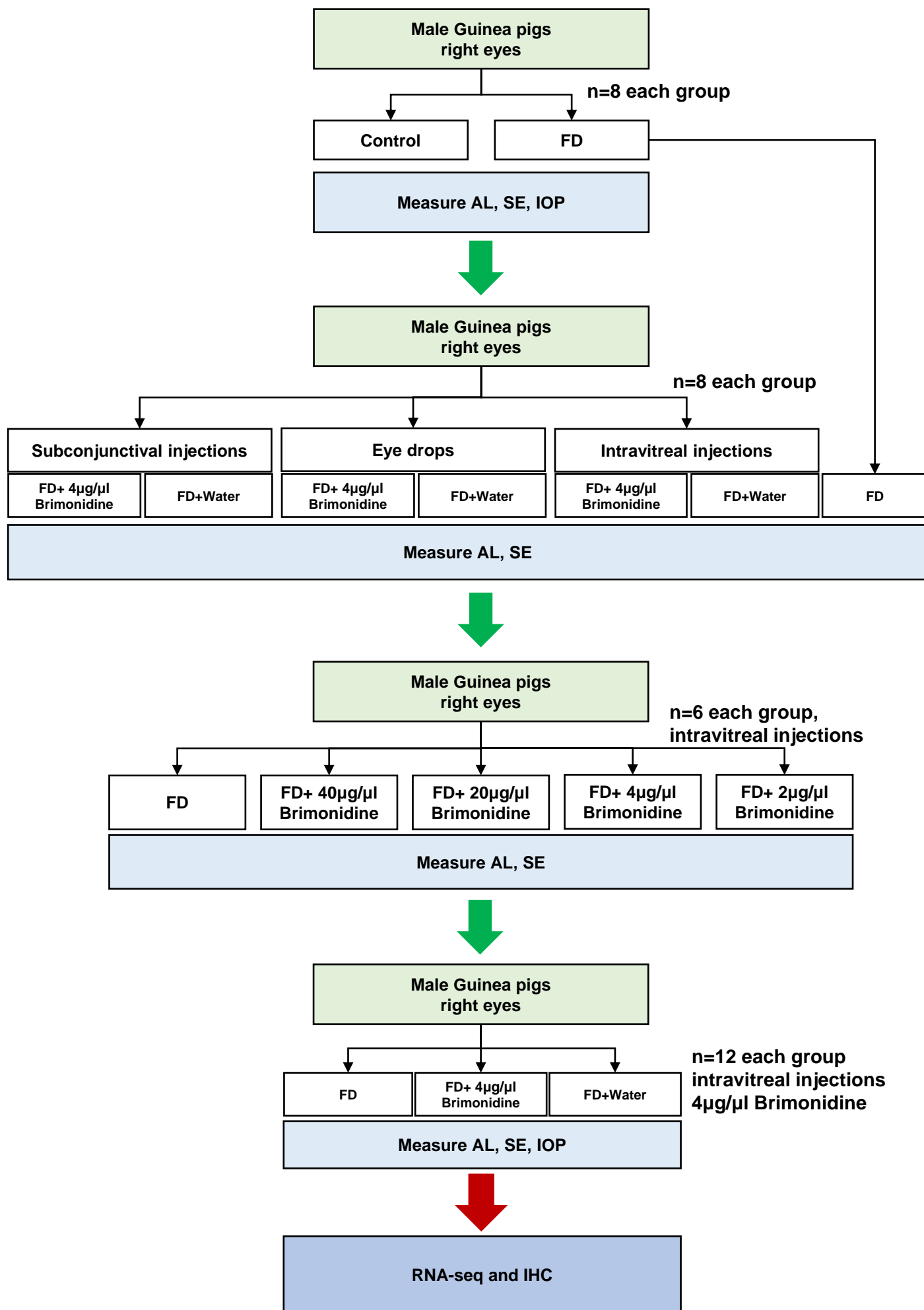

Supplement: Supplementary file 2 — Additional file 2: Supplementary Figure 2. Flow chart of experimental design. [file 40662_2021_248_MOESM2_ESM.pdf]

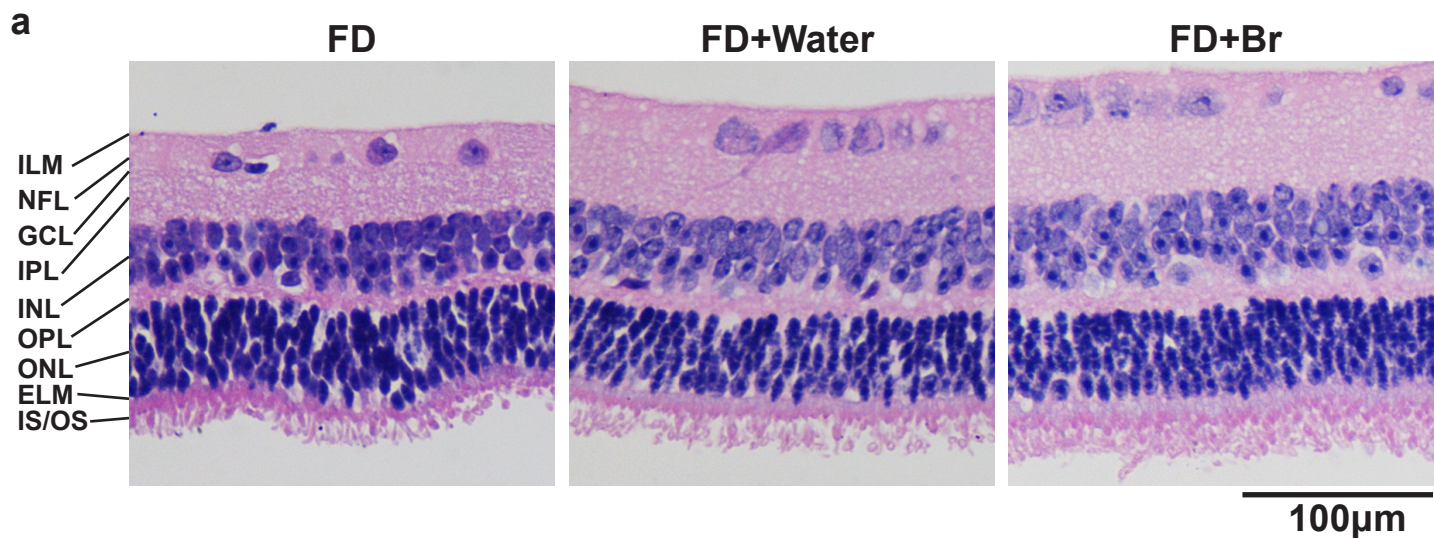

**b**

Adrenergic signaling Related Gene set

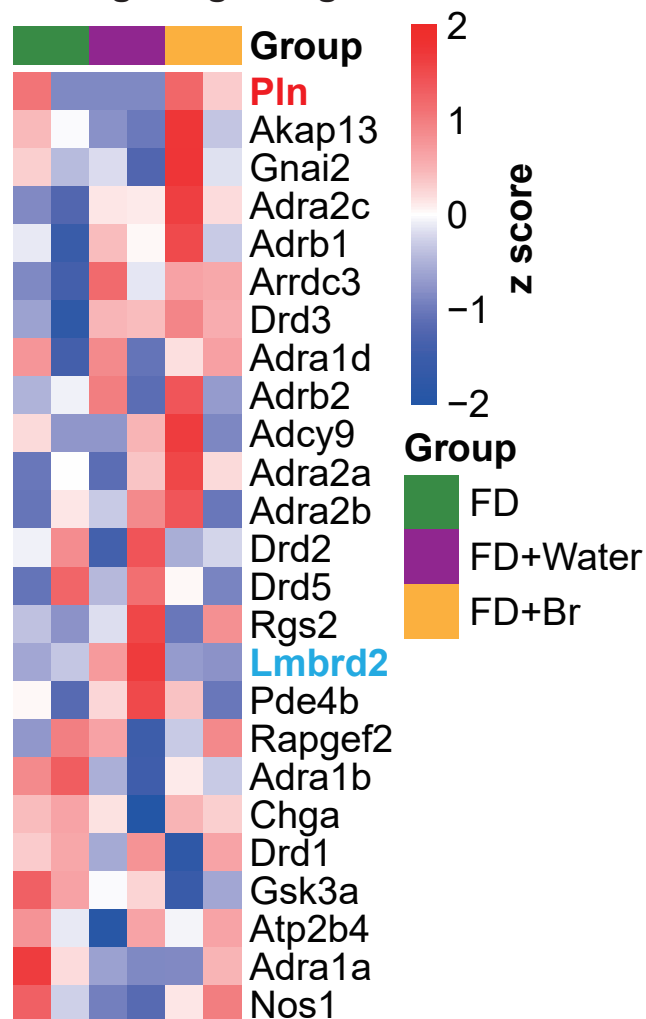

**c**

Cholinergic signaling Related Gene set

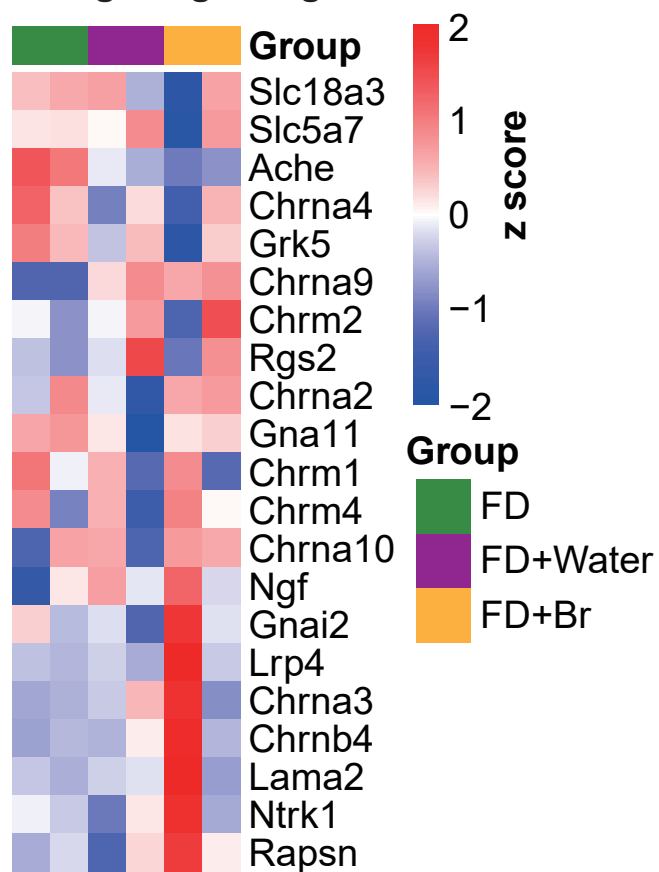

Supplement: Supplementary file 3 — Additional file 3: Supplementary Figure 3. Labelled retinal layers (ILM, internal limiting membrane. NFL, nerve fiber layer. GCL, ganglion cells layer. IPL, inner plexiform layer. INL, inner nuclear layer. OPL, outer plexiform layer. ONL, outer nuclear layer. ELM, external limiting membrane. IS/OS, inner segment/outer segment). Adrenergic and cholinergic signaling-related gene set. [file 40662_2021_248_MOESM3_ESM.pdf]
